# A rare case of a giant arterio-venous fistula (AVF) following metastatic choriocarcinoma conditioning pulmonary embolism: multimodal transcatheter embolization using a simultaneous transarterial and transvenous approach

**DOI:** 10.1186/s42155-018-0039-8

**Published:** 2018-11-20

**Authors:** Massimo Venturini, Alice Bergamini, Anna Colarieti, Micaela Petrone, Paolo Marra, Emanuela Rabaiotti, Giorgia Mangili, Massimo Candiani, Alessandro Del Maschio, Francesco De Cobelli

**Affiliations:** 1 Department of Radiology, San Raffaele Scientific Institute, Vita-Salute San Raffaele University, Via Olgettina 60, 20132 Milan, Italy; 2 Department of Gynaecology, San Raffaele Scientific Institute, Milan, Italy; 3 Vita-Salute San Raffaele University, Milan, Italy

**Keywords:** Choriocarcinoma metastases, Pulmonary embolism, Arteriovenous fistula, Embolization, Embolic agents, Amplatzer vascular plug

## Abstract

**Background:**

Choriocarcinoma is a highly malignant tumor but with a good prognosis due to the valid response to systemic chemotherapy. We present a case of a young woman affected by a giant pelvic arterio-venous fistula following a metastatic gestational choriocarcinoma, conditioning metrorrhagia and pulmonary embolism, successfully treated by multimodal transcatheter embolization, using a simultaneous transarterial and transvenous approach.

**Case presentation:**

In a young patient affected by choriocarcinoma and metrorrhagia, a computed tomography showed a giant arterio-venous fistula, pulmonary metastases and embolism. A transfemoral diagnostic arteriography showed a giant arterio-venous fistula sustained by right and left hypogastric arteries with early opacification of the right gonadal vein and of the inferior vena cava. A transarterial embolization of the distal branches of hypogastric arteries with poly-vinyl-alcohol particles, coils and Squid was performed. A transfemoral phlebography of the right gonadal vein showed multiple thrombi, responsible of the pulmonary embolism. An Amplatzer plug via trans-jugular was finally placed at the confluence of the gonadal vein in the vena cava, to reduce arterio-venous fistula out-flow and to occlude the vein, preventing further episodes of pulmonary embolism. Metrorrhagia progressively disappeared. A second transarterial embolization combined with a complete response to systemic chemotherapy determined arterio-venous fistula resolution.

**Conclusions:**

This was a very rare case of a giant pelvic arterio-venous fistula following choriocarcinoma in a patient symptomatic for metrorrhagia with an accidental finding of pulmonary embolism at computed tomography. A transcatheter embolization was successfully performed with different embolic materials, using a simultaneous transarterial and transvenous approach: the goal was not only to obtain metrorrhagia resolution but also to avoid a massive pulmonary embolism, a potential life threatening condition, in a young woman affected by a highly malignant tumor but with a good prognosis.

## Background

Gestational choriocarcinoma is a highly malignant tumor characterized by abnormal trophoblastic hyperplasia, absence of chorionic villi, hemorrhage, and necrosis. Myometrium and vascular invasion are typical features, resulting in spread to distant sites, most commonly to the lungs (Mangili et al., 2014). It affects one in 40 000 pregnancies and one in 40 hydatidiform moles and can determine massive uterine bleeding and arterio-venous fistula (AVF), in selected cases treated with transcatheter embolization (Alturkistani et al., 2016; Wang et al., 2017). Choriocarcinoma-associated pulmonary thromboembolism represents another complication (Zhu et al., 2016) and thrombolysis a potential therapeutic option (Yang & Peng, 2017). Despite this aggressive behaviour, it has a good prognosis due to the valid response to systemic chemotherapy (Wang et al., 2017; Yang & Peng, 2017). This can be monitored by serum beta Human Chorionic Gonadotropin (β-HCG) measurements, secreted by the tumor. We present a case of a young woman affected by a giant pelvic AVF following a metastatic gestational choriocarcinoma, conditioning metrorrhagia and pulmonary embolism, successfully treated by multimodal transcatheter embolization, using a simultaneous transarterial and transvenous approach.

## Case presentation

**Fig. 1:**
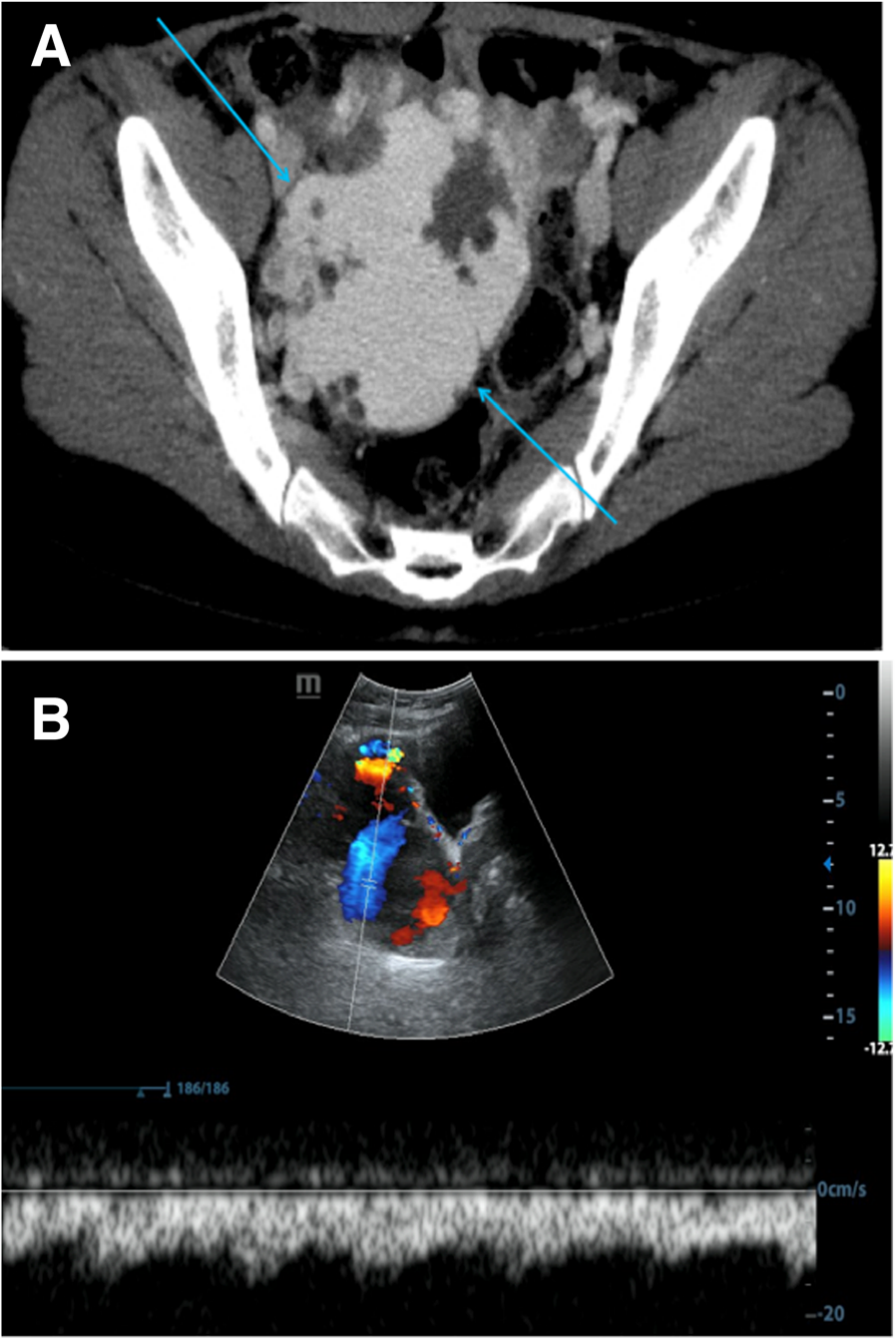
Contrast-enhanced MDCT shows **(a)** a suspicious giant pelvic AVF (arrows), **(b)** confirmed by Color Doppler

**Fig. 2:**
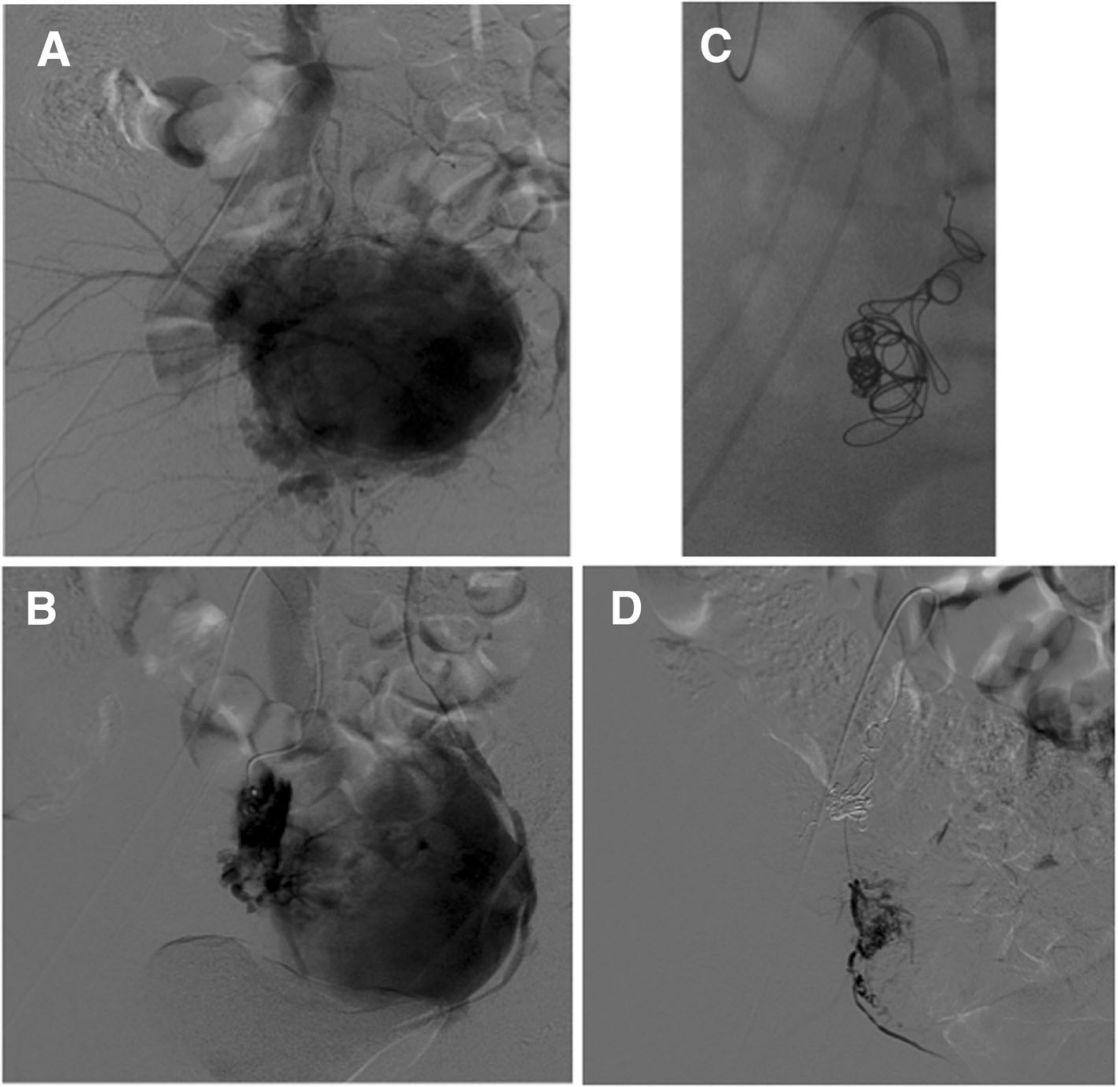
Diagnostic arteriography from the right hypogastric artery shows **(a)** a giant AVF with early opacification of the right gonadal vein and the inferior vena cava. DSA performed with a microcatheter shows peripheral arterial branches sustaining the fistula **(b)**, embolized using coils **(c)** and PVA particles **(d)**

**Fig. 3:**
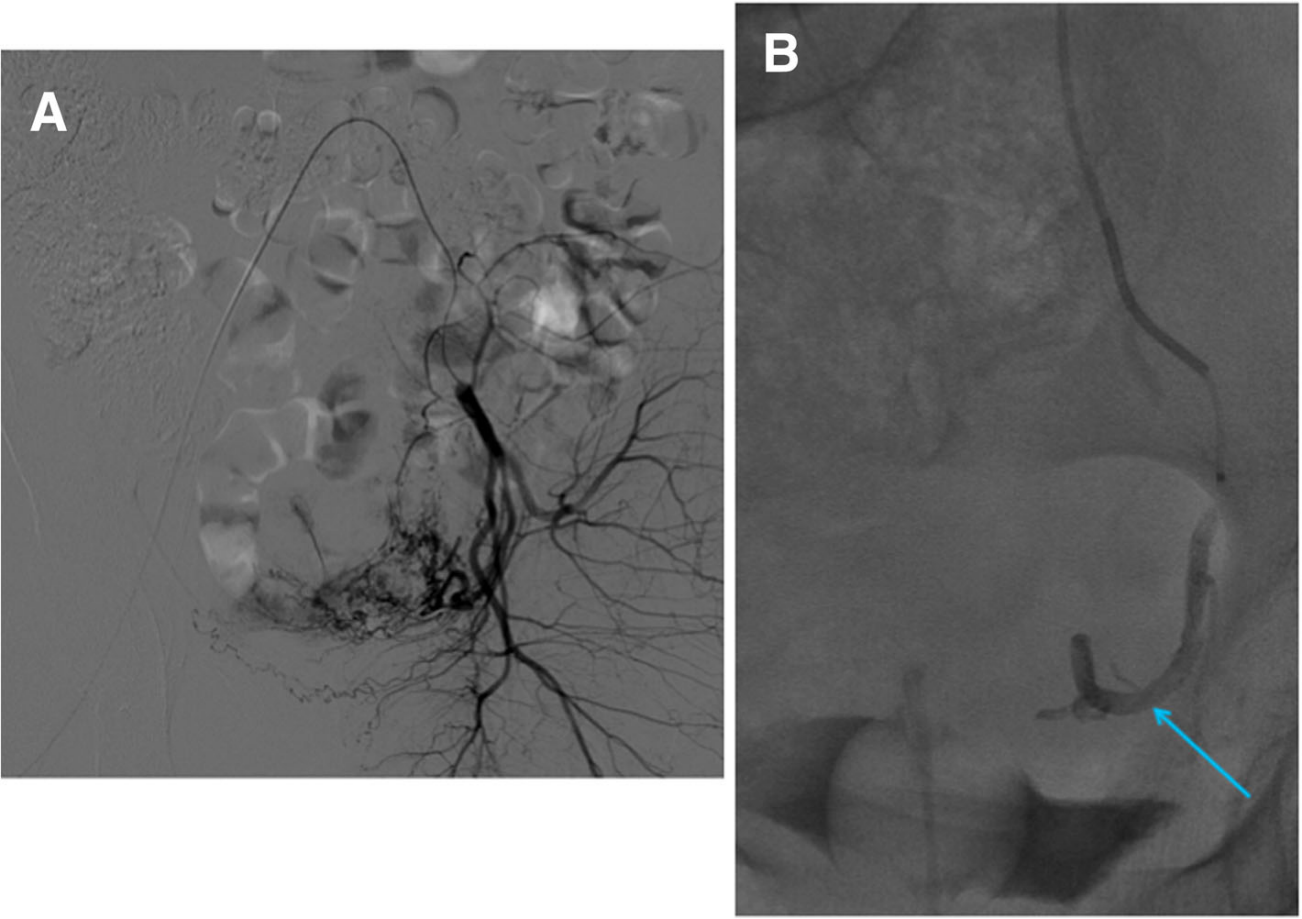
DSA shows branches of the left hypogastric artery sustaining the AVF **(a)**, embolized also using Squid (arrow) **(b)**

**Fig. 4:**
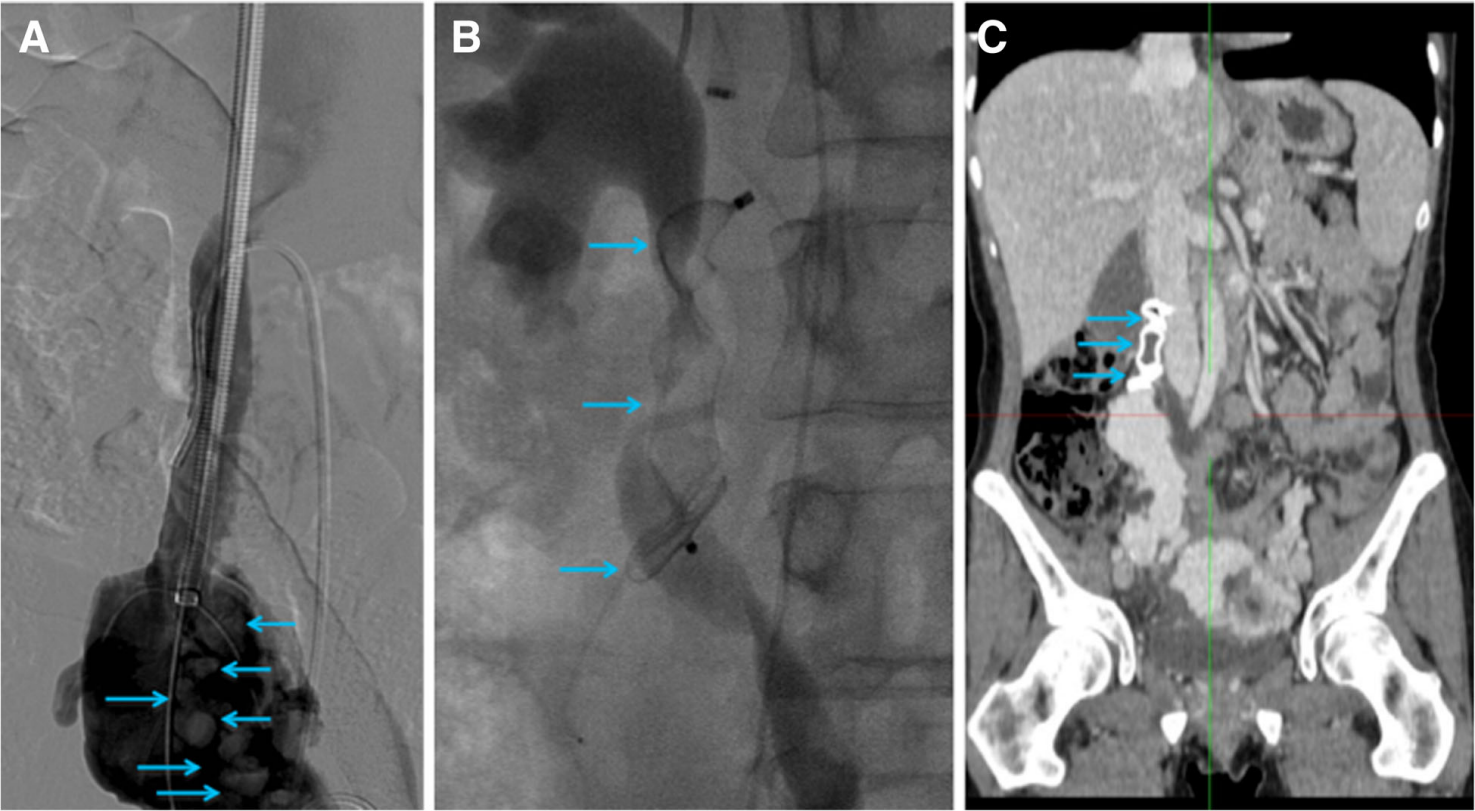
Diagnostic phlebography via trans-femoral of the right gonadal vein shows **(a)** multiple thrombi (arrows). Amplatzer plug placement (arrows) via trans-jugular to occlude the right gonadal vein **(b)** to simultaneously reduce AVF-outflow and avoid pulmonary embolism. Contrast-enhanced MDCT after 24 h confirms **(c)** the correct placement of the plug (arrows)

**Fig. 5:**
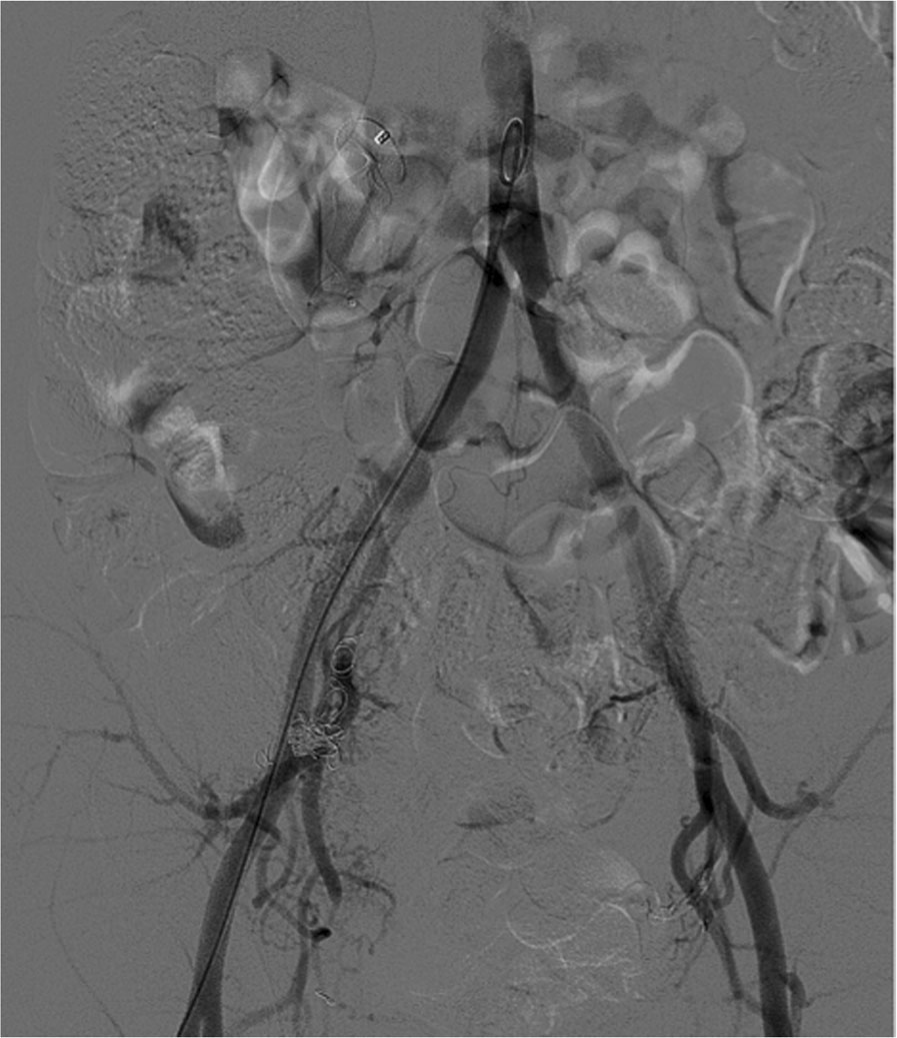
After the second arterial embolization performed 6 months later, the final aortography shows the complete AVF occlusion

A 37-year-old woman was referred to our institute (Gynaecology Department) due to persistent metrorrhagia and raised serum β-HCG levels (126031 mU/ml). Patient gynaecological and obstetrical history was characterized by one prior term birth in 2012 and a spontaneous miscarriage at seven gestational weeks in 2016. The suspicion of gestational choriocarcinoma was raised as a highly vascularized uterine mass was detected at computed tomography. Gestational Choriocarcinoma is a highly malignant neoplasm of trophoblastic origin, characterized by rapid growth and high tendency to develop hematogenous metastases. Diagnosis is more commonly based on β-HCG serum levels and clinical presentation rather than on histopatological analysis (due to the high risk of bleeding following bioptical procedures). Thanks to its high chemosensitivity, gestational choriocarcinoma is usually associated with a good prognosis and high cure rates. The patient was submitted to a total body triphasic contrast-enhanced Multi Detector Computed Tomography (MDCT) confirming the presence of choriocarcinoma, but also showing a giant pelvic aneurysm suspecious for AVF (Fig. 1), lung metastases and pulmonary thrombo-embolisms. The diagnosis of AVF was confirmed by a Color Doppler Ultrasound examination showing a typical arterialized, low-resistance blood flow of the pelvic veins (Fig. 1). The case was discussed within a multidisciplinary gynaecological and radiological meeting. Following this, an angiography was planned in order to confirm the AVF diagnosis and to perform an embolization to stop the bleeding trying to occlude the fistula despite its large size. The decision on the opportunity to place a filter to prevent further episodes of pulmonary embolism was postponed until diagnostic angiograpy and embolization were completed. In an emergency setting, the patient was submitted to a diagnostic angiography initially using a right femoral transarterial and right femoral transvenous approach. Diagnostic arteriography confirmed the presence of a giant AVF sustained by branches of both hypogastric arteries with early opacification of the right gonadal vein and the inferior vena cava (Fig. 2). After selective catheterization of right (Fig. 2) and left (Fig. 3) hypogastric arteries, using a coaxial microcatheter (Carnelian 2.2, Tokai, Medical Products, Sarayashiki Taraga Kasugay-city, Japan), the afferent branches to AVF were subsequently embolized using first detachable coils (Interlock, Boston Scientific, Natick, MA, USA) of variable diameter (6–14 mm) and length (10–40 cm), after poly vinyl alcohol (PVA) particles (Contour Embolization particles 500–710 μ, Boston Scientific, Natick, MA, USA) and finally also an ethylene-vinyl alcohol copolymer (EVOH)-based liquid embolic agent (Squid-peri 12, Emboflu, Gland, Switzerland) in order to reduce AVF in-flow (Figs. 2 and 3). A transfemoral phlebography with selective catheterization of the right gonadal vein showed multiple thrombi (Fig. 4), leading to the pulmonary embolism previously detected at the contrast-enhanced MDCT. Using a right transjugular approach, an Amplatzer plug was finally placed at the confluence of the right gonadal vein in the vena cava (Fig. 4), not only to reduce AVF out-flow but also to occlude the right gonadal vein, preventing further episodes of pulmonary embolism. Metrorrhagia almost disappeared after the procedure. A contrast-enhanced MDCT examination performed 24 h after the embolization confirmed the correct placement of the plug (Fig. 4) and the significant reduction in volume and enhancement of the AVF. No further pulmonary embolism was demonstrated at MDCT performed during follow-up. A second transarterial embolization using the same embolic agents (PVA particles, coils and Squid) was performed six months later. The second embolization, combined with a complete response to systemic chemotherapy confirmed by β-HCG levels normalization with disappearance of pulmonary metastases, determined the complete AVF resolution (Fig. 5). Currently the patient is aymptomatic and enjoys full well-being of health.

## Conclusions

AVFs following gestational choriocarcinoma, treated with embolization (Alturkistani et al., 2016) or conservatively (Kim et al., 2013), were previously described. In the present case, a young woman symptomatic for metrorrhagia but without chest pain or dyspnea was admitted to our institute. Contrast-enhanced MDCT showed a metastatic choriocarcinoma (confined to the lung) and a giant AVF conditioning not only metrorrhagia but also pulmonary embolism, with diagnostic angiographic evidence of multiple thrombi within the right gonadal vein. Hence the choice to perform a simultaneous transarterial and transvenous embolization. Different embolic agents were used to embolize distal branches of hypogastric arteries obtaining a progressive reduction of AVF in-flow: PVA particles were mainly used for the nidus (Sheth et al., 2017), while coils (Edward Garrett Jr & Mack, 2017) and Squid (Akmangit et al., 2014) for the AVF feeding branches. As Onyx, Squid is an EVOH-based liquid embolic agent well known in cerebral field until now less used in abdominal district. Differently from Onyx, Squid is also available in the new formulation Squid-12, used in our case and characterized by lower density and viscosity, able to improve vascular penetration and distal distribution compared to formulation Onyx- and Squid-18. Then using a right transjugular approach, an Amplatzer plug (Tapping et al., 2011) was placed at the confluence of the right gonadal vein in the vena cava not only to reduce AVF out-flow but also to definitively occlude the right gonadal vein to avoid further episodes of pulmonary embolism. The choice to prefer a jugular approach instead of transfemoral to place the plug was due to the more favourable angle. The decision for a venous plug to prevent embolization and ensure a stable protection was made during the procedure considering the detection at phlebography of multiple thrombi in the right gonadal vein. As alternative to plug, the placement of a retrievable inferior vena cava filter in supra-renal position was not considered for the following reasons: small thrombus could pass through the filter legs, retrievable inferior vena cava filters, particularly in supra-renal position, can give complications (Venturini et al., 2015) and finally plug occluding the vein was able not only to avoid further episodes of pulmonary embolism but also to stop AVF out-flow. Despite the simultaneous transarterial and transvenous approach combined with the multiple embolic agents used, the large size of the AVF doesn’t allow its occlusion in a single procedure. However a significant shrinkage sufficient to ensure metrorrhagia resolution was obtained. Definitive AVF occlusion was achieved 6 months later due to a second transarterial embolization procedure combined with the progressive efficient action of systemic chemotherapy.

In conclusion, this was a rare case of a giant AVF following gestational choriocarcinoma in a young patient symptomatic for metrorrhagia, without dyspnea or chest pain, with the accidental finding of pulmonary embolism. A transcatheter embolization in emergency was successfully performed with different embolic materials, using a simultaneous transarterial and transvenous approach: the goal was not only to obtain metrorrhagia resolution but also to avoid a massive pulmonary embolism, a potential life threatening condition, in a young woman affected by a highly malignant tumor but characterized by a good prognosis.

## Abbreviations

AVF: Arteriovenous fistula
EVOH: Ethylene-vinyl alcohol copolymer
MDCT: Multi detector computed tomography
PVA: Poly vinyl alcohol
β-HCG: Beta human chorionic gonadotropin
