# Correction to: A rare case of a giant arterio-venous fistula (AVF) following metastatic choriocarcinoma conditioning pulmonary embolism: multimodal transcatheter embolization using a simultaneous transarterial and transvenous approach

**DOI:** 10.1186/s42155-020-0100-2

**Published:** 2020-03-02

**Authors:** Massimo Venturini, Alice Bergamini, Anna Colarieti, Micaela Petrone, Paolo Marra, Emanuela Rabaiotti, Giorgia Mangili, Massimo Candiani, Alessandro Del Maschio, Francesco De Cobelli

**Affiliations:** 1 Department of Radiology, San Raffaele Scientific Institute, Vita-Salute San Raffaele University, Via Olgettina 60, 20132 Milan, Italy; 2 Department of Gynaecology, San Raffaele Scientific Institute, Milan, Italy; 3 Vita-Salute San Raffaele University, Milan, Italy

**Correction to: CVIR Endovasc (2018) 1:30**

**https://doi.org/10.1186/s42155-018-0039-8**

‘In the published article (Venturini et al. 2018) the statement under the subheading ‘Consent for publication’ is incorrect.

“Not applicable”

should read:

“Informed consent for publication of this case report and its accompanying images has been obtained from the patient”

## References

[CR1] Venturini M, Bergamini A, Colarieti A, Petrone M, Marra P, Rabaiotti E, Mangili G, Candiani M, Del Maschio A, De Cobelli F (2018). A rare case of a giant arterio-venous fistula (AVF) following metastatic choriocarcinoma conditioning pulmonary embolism: multimodal transcatheter embolization using a simultaneous transarterial and transvenous approach. CVIR Endovasc.

